# Evidencing the Role of Erythrocytic Apoptosis in Malarial Anemia

**DOI:** 10.3389/fcimb.2016.00176

**Published:** 2016-12-09

**Authors:** Paulo R. R. Totino, Cláudio T. Daniel-Ribeiro, Maria de Fátima Ferreira-da-Cruz

**Affiliations:** Laboratory of Malaria Research, Instituto Oswaldo Cruz, Fundação Oswaldo CruzRio de Janeiro, Brazil

**Keywords:** malaria, anemia, apoptosis, red blood cells, phagocytosis

## Abstract

In the last decade it has become clear that, similarly to nucleated cells, enucleated red blood cells (RBCs) are susceptible to programmed apoptotic cell death. Erythrocytic apoptosis seems to play a role in physiological clearance of aged RBCs, but it may also be implicated in anemia of different etiological sources including drug therapy and infectious diseases. In malaria, severe anemia is a common complication leading to death of children and pregnant women living in malaria-endemic regions of Africa. The pathogenesis of malarial anemia is multifactorial and involves both ineffective production of RBCs by the bone marrow and premature elimination of non-parasitized RBCs, phenomena potentially associated with apoptosis. In the present overview, we discuss evidences associating erythrocytic apoptosis with the pathogenesis of severe malarial anemia, as well as with regulation of parasite clearance in malaria. Efforts to understand the role of erythrocytic apoptosis in malarial anemia can help to identify potential targets for therapeutic intervention based on apoptotic pathways and consequently, mitigate the harmful impact of malaria in global public health.

## Introduction

Kerr and colleagues introduced the term *apoptosis* in 1972 to designate a physiologic process of programmed cell death implicated in normal maintenance of tissue cell population as well as in development of pathologies such as cancer. Since then, an enormous number of studies focusing on apoptosis have been published. These studies have focused on apoptosis of nucleated cells, whose dramatic changes occurring in the nucleus, i.e., chromatin condensation and DNA fragmentation (Kerr et al., 1972; Wyllie, 1980; Totino et al., 2006), labeled apoptosis as a nucleus-dependent stereotyped process. However, pioneer studies with experimentally enucleated cells demonstrated that typical cytoplasmic and cell membrane features of apoptosis, such as membrane blebbing, cell shrinkage, loss of mitochondrial transmembrane potential, and exposure of phosphatidylserine can be induced in the absence of a nucleus (Jacobson et al., 1994; Schulze-Osthoff et al., 1994; Castedo et al., 1996), raising the possibility that apoptosis could also determine the life span of physiologically enucleated cells, i.e., the red blood cells (RBC).

Indeed, even the lack of organelles in RBCs, including those directly implicated in apoptosis induction (i.e., mitochondria and endoplasmic reticulum), does not impair activation of the cytoplasmic machinery present in the cell that coordinates the apoptotic process (Bratosin et al., 2001; Lang et al., 2012). Although the pathways leading to apoptosis in RBC are not well known, they have been shown to be initiated by an increase in cytosolic Ca^2+^ due to activation of Ca^2+^ permeable non-selective cation channels in the cell membrane, which can be triggered by a variety of xenobiotics and endogenous substances (Lang et al., 2012). In turn, influx of Ca^2+^ leads to (1) activation of caspases and calpains, which participate in cell disassembly and formation of cell membrane blebbing; (2) cell shrinkage through exit of K^+^ via Ca^2+^-sensitive K^+^ channel; and (3) stimulation of cell membrane scrambling, resulting in phosphatidylserine (PS) exposure at the cell surface (Gao et al., 2012; Lang et al., 2012). This last event is critical for elimination of apoptotic cells, before cell disintegration, and subsequent release of cytoplasmic proinflammatory mediators, because PS works as an “eat me” signal for phagocytic cells that operate in an anti-inflammatory manner (Wu et al., 2006).

Similarly to apoptosis of nucleated cells, apoptosis of erythrocytes participates in the body homeostasis through physiological clearance of aged cells. It is estimated that 360 billion of senescent erythrocytes undergo apoptotic processes and are removed every day by the spleen, avoiding systemic complications related to intravascular hemolysis (Bratosin et al., 1998, 2009). On the other hand, apoptosis is also believed to contribute to anemia and vascular disorders associated with clinical conditions in which excessive rates of apoptotic RBCs are documented, including diabetes, renal insufficiency, sickle-cell anemia, chronic lead exposure (Lang et al., 2002; Myssina et al., 2003; Calderón-Salinas et al., 2011; Aguilar-Dorado et al., 2014) as well as infectious diseases such as sepsis and mycoplasmosis (Kempe et al., 2007; Felder et al., 2011).

In view of the pathophysiological significance of erythrocytic apoptosis, our group has attempted to study this erythrocytic process in malaria, a vector-borne infection caused by protozoa of the genus *Plasmodium* that infect RBCs and induce strong hematologic and vascular disturbances (Anstey et al., 2009; Grau and Craig, 2012; Carvalho et al., 2014). Our studies have shown that induction of apoptosis is not limited to parasitized RBCs (pRBCs) but also occurs in non-parasitized RBCs (nRBCs), pointing to an involvement of both pRBC and nRBC in the pathogenesis of malaria through deflagration of suicide processes (Totino et al., 2009, 2011, 2013, 2014). Thus, the present review covers evidence implicating erythrocytic apoptosis in the pathogenesis of severe anemia, a common complication of malaria that represents an important public health concern strongly related to mortality in children and pregnant women living in malaria-hyperendemic regions of sub-Saharan Africa (Schantz-Dunn and Nour, 2009; Muoneke et al., 2012).

## Malarial anemia and RBC removal

It should not be a surprise that anemia is one of the most common disorders associated with malaria, because *Plasmodium* parasites develop an intraerythrocytic schizogony process culminating in lysis of RBCs. In non-immune children and adults infected with *P. falciparum*, the degree of anemia may correlate with parasitemia (Phillips and Pasvol, 1992; Biemba et al., 2000; Menendez et al., 2000) and in most experimental rodent malaria, acute anemia is a consequence of the rupture of high percentages of pRBCs (Lamikanra et al., 2007). Nevertheless, it is known that destruction of pRBCs alone cannot account for anemia severity in malaria patients (Ekvall, 2003; Akinosoglou et al., 2012). Acute anemia can be observed in both experimental and human infections presenting low parasitemia and even after antimalarial treatment and clearance of parasites, anemia can persist or worsen (Phillips et al., 1986; Camacho et al., 1998; Carvalho et al., 2003; Helleberg et al., 2005; Evans et al., 2006). In view of these facts, both ineffective production of RBCs by the bone marrow and premature elimination of nRBC have been postulated as important determinants of malaria anemia pathogenesis (Jakeman et al., 1999; Casals-Pascual et al., 2006; Evans et al., 2006; Thawani et al., 2014).

The extent of RBC elimination in malaria was reported in studies estimating that, for every pRBC, up to 32 nRBC are removed from circulation (Jakeman et al., 1999; Collins et al., 2003; Evans et al., 2006). In addition, there is evidence supporting that nRBC removal is mediated by phagocytic activity rather than intravascular hemolysis (Phillips et al., 1986; Abdalla, 1988; Goka et al., 2001; La Raja, 2002; Satpathy et al., 2004; Evans et al., 2006), highlighting the mononuclear phagocytic reticuloendothelial system as an important mechanism of malarial anemia pathogenesis. Indeed, besides directly participate in malarial anemia by uptake of both pRBCs and nRBCs, macrophages may also interfere with the erythropoietic process by limiting systemic bioavailability of recycled iron, the main source of which is erythrophagocytosis occurring in spleen and bone marrow (Beaumont and Delaby, 2009; Spottiswoode et al., 2014). However, the pathways underlying clearance of RBCs in malarial anemia remain elusive and therefore, apoptosis as a well-known mechanism of cell depletion participating in cytopenia of several pathologies could mediate elimination of nRBCs.

## Malaria and erythrocytic apoptosis inducers

Studies conducted by Dondorp et al. (1999) with *P. falciparum*-infected patients demonstrated that similarly to pRBCs, in which parasites induces drastic changes on the host cell membrane, nRBCs also present reduced deformability correlating with anemia severity. This phenomenon, also recorded in nRBC from *P. falciparum in vitro* culture and *P. yoelii*-infected mice (Paul et al., 2013; Huang et al., 2014), has been related to adsorption of malaria parasite antigens on the cell surface (Layez et al., 2005; Omodeo-Salè et al., 2005) and interestingly, more recently identified as a hallmark of erythrocytic apoptosis (Mindukshev et al., 2013), raising the possibility that antigens of malaria parasites have proapoptotic proprieties on RBCs.

Microbes in general could affect proapoptotic pathways in host cells****. Gram-positive and gram-negative bacteria, as well as protozoan parasites, have been shown to manipulate apoptotic processes of both infected and non-infected host nucleated cells via pathogen antigens that act as apoptosis regulatory molecules through multifarious pathways, impacting on the outcome of disease (Ulett and Adderson, 2006; Rodrigues et al., 2012). A diversity of antigens produced by pathogenic bacteria such as enterotoxins, hemolysins, lethal toxins, lipotechoic acid, and lipopolysaccharide, have the ability to promote apoptosis in several types of host cells including T and B lymphocytes, dendritic cells, monocytes, macrophages, neutrophils, endothelial cells, epithelial cells (Ulett and Adderson, 2006; Carrero and Unanue, 2012). Similarly, malaria toxins glycosylphosphatidylinositol and hemozoin purified from *P. falciparum* showed proapoptotic activity in cardiomyocytes and erythroblasts, respectively (Wennicke et al., 2008; Lamikanra et al., 2009). Moreover, such apoptogenic effect of parasite factors was recorded by incubating brain vascular endothelial and neuroglia cells with *P. falciparum*-pRBC conditioned medium (Wilson et al., 2008), and induction of apoptosis in lung endothelial cells can additionally take place by physical contact due to pRBC adhesion phenomenon (Pino et al., 2003).

There are no studies further exploring the proapoptotic potential of plasmodial antigens in erythrocytic apoptosis. However, it was already reported that in *P. falciparum in vitro* cultures nRBCs are marked by PS externalization (Koka et al., 2007; Pattanapanyasat et al., 2010), and in our previous study with BALB/c mice infected by the lethal *P. yoelii* 17XL parasites, increased levels of nRBC apoptosis correlated with hiperparasitemia (Totino et al., 2013), indirectly suggesting that as do bacterial toxins (listeriolysin from *Listeria monocytogenes* and hemolysin from *Vibrio parahaemolyticus*), peptidoglycan, and *Schistosoma mansoni* egg antigens (Lang et al., 2004b; Föller et al., 2007; Kasinathan and Greenberg, 2010; Jilani et al., 2012; Abed et al., 2013), plasmodial antigens can play a role in induction of erythrocytic apoptosis.

Additionally, several host factors have the potential to modulate erythrocytic apoptosis during malaria (Lang and Lang, 2015). They may comprise: (1) proapoptotic factors whose increased levels are associated with malarial severity, such as soluble Fas ligand (sFasL), anti-erythrocyte antibodies, hematin, granzyme B, and oxidative stress (Das et al., 1993; Waitumbi et al., 2000; Hermsen et al., 2003; Issifou et al., 2003; Dalko et al., 2015) and; (2) antiapoptotic factors, i.e., erythropoietin, vitamin E, and nitric oxide, that show either reduced levels or low bioavability in malaria, and consequently, could contribute to amplify erythrocytic apoptosis induction (Burgmann et al., 1996; Kulkarni et al., 2003; Sobolewski et al., 2005).

It is also noteworthy that several routine therapeutic drugs present proapoptotic properties on erythrocytes, which has been linked to drug-associated anemia (Lang and Lang, 2015). *In vitro* studies performed by Alzoubi et al. (2014a,b) showed that the antimalarial drugs artesunate and lumefantrine have proapoptotic effect on erythrocytes, possibly through generation of oxidative stress, a feature already reported for both drugs (Xie et al., 2005; Abolaji et al., 2013). Thus, the proapoptotic effect of artesunate and lumefantrine could explain the reduction of erythrocytic blood parameters reported in healthy rats and humans (Xie et al., 2005; Kongpatanakul et al., 2009) as well as in occurrence of acute anemia in malarial patients following antimalarial drug treatment (De Nardo et al., 2013; Raffray et al., 2014; Sowunmi et al., 2015).

## Removing nRBCs in malaria by apoptosis

Although it is known that sensitization of nRBCs by antibodies and complement system contributes to malarial anemia pathogenesis ((Daniel-Ribeiro et al., 1986); (Daniel-Ribeiro and Zanini, 2000; Waitumbi et al., 2000), there is evidence indicating that the elimination of nRBCs could also occur in an opsonin-independent manner. For instance, a lack of correlation between RBC sensitization and anemia degree has been reported in some studies with malarial patients (Abdalla, 1986; Merry et al., 1986) and a shortened lifespan of nRBCs was observed during *P. yoelii* induced-anemia in a B cell-deficient environment provided by severe combined immunodeficient mice (Salmon et al., 1997). In addition, in a recently described novel model of severe malarial anemia presenting low parasitemia, C3^−/−^ mice still developed acute anemia following *P. berghei* infection (Harris et al., 2012).

The participation of a nonopsonic phagocytosis in nRBC elimination and its relation to apoptosis can be sustained by data concerning the scavenger receptor CD36 and PS. CD36 is a well-known phagocytic receptor for apoptotic cells that acts via recognition of PS on the apoptotic cell surface (Greenberg et al., 2006). Experiments with both monoclonal anti-CD36 antibodies and CD36-deficient rodent macrophages have shown that CD36 plays also a role in phagocytic uptake of RBC in malaria (McGilvray et al., 2000; Patel et al., 2004; Ayi et al., 2005). In these experiments, the contribution of parasite antigens with affinity to CD36 (e.g., *P. falciparum* erythrocyte membrane protein 1—PfEMP-1) on the pRBC surface cannot be ruled out, since they were performed with pRBC. Nevertheless, intraerythrocytic plasmodia development progressively induces PS exteriorization on pRBC as a result of non-selective cation channel activation in the host RBC membrane (Eda and Sherman, 2002; Lang et al., 2004a), indicating that pRBC could be removed via PS.

Importantly, PS has been recognized as a key molecule mediating erythrophagocytosis, as *in vitro* treatment with annexin V or PS-containing liposomes is able to inhibit phagocytosis of senescent or oxidatively stressed RBC, an effect also achieved with the use of anti-CD36 antibodies (Bratosin et al., 1997; Tait and Smith, 1999; Mandal et al., 2002). In a similar way, phagocytosis of RBC from uremic patients, with increased levels of PS-exposing RBC with great propensity for phagocytosis, was also inhibited after preincubation of macrophages with glycerophosphorylserine, a structural derivate of PS (Bonomini et al., 2001), indicating that PS can mediate shortened life span of RBC in pathological conditions in which anemia is a common feature. In this context, anemia has been associated with the rates of PS-exposing RBC in peritoneal dialysis and sepsis patients (Bi et al., 2006; Shi et al., 2013) as well as with erythrophagocytosis mediated by PS in rats treated with lead or naphthoquinone—two inducers of erythrocytic apoptosis (Noh et al., 2010; Jang et al., 2011).

Hence, PS exposure on nRBCs induced to apoptosis during malaria could also be involved in anemia development, as suggested initially by our studies on lethal *P. yoelii* experimental infection, in which the levels of PS-exposing nRBCs were increased during the stage of infection marked by an acute anemia (Totino et al., 2010). Both erythrophagocytosis and PS-exposing RBCs are increased in *P. falciparum* infected-children presenting severe anemia as compared to those with uncomplicated malaria (Fendel et al., 2010). Apoptosis can also be induced by incubating RBCs from healthy individuals with plasma from *P. falciparum* patients, an effect that is not observed with plasma from *P. vivax* infections, which, comparatively to *P. falciparum*, are less prone to lead to severe disease (Totino et al., 2014). Furthermore, since CD36 is a key molecule in the RBC clearance process, resistance to severe malarial anemia reported in children with a non-sense CD36 mutation can be explained, in part, by a deficiency in phagocytosis of apoptotic nRBCs mediated by CD36-PS interaction (Pain et al., 2001; Chilongola et al., 2009).

Recently, additional evidence for the contribution of PS to malarial anemia was reported in a study of mice infected with the nonlethal (17XNL) *P. yoelii* (Fernandez-Arias et al., 2016). In this model, as observed by our group with the lethal (17XL) strain *P. yoelii* (Totino et al., 2010), there was a significant increase in the levels of PS-exposing nRBCs. In addition, it was shown that *P. yoelii* infection induces autoantibodies against PS exposed on nRBCs and that these antibodies enhance anemia when transferred to infected mice, an effect related to *in vitro* nRBC phagocytosis. Importantly, anti-PS antibodies were also detected in sera from *P. falciparum*-infected patients and the highest levels were associated with late postmalarial anemia in nonimmune travelers. Therefore, besides participating in the pathogenesis of malarial anemia by directly promoting RBC elimination via the innate immune receptor CD36, PS may also contributes antigenically as a target of acquired immune response, a novel described autoimmune phenomenon in malaria whose source needs further investigation.

Another cell event that links erythrocytic apoptosis and anemia is a change in CD47 expression. CD47 is a ubiquitous glycoprotein expressed on the cell surface that was identified as a “marker of self” by suppressing phagocytosis of normal viable cells through interaction with signal-regulatory protein alpha (SIRPα), which operates as a inhibitory receptor on phagocytes (Sosale et al., 2015). Initially identified in studies demonstrating a rapid *in vivo* clearance of CD47-deficient RBCs and an increased *in vitro* phagocytosis of normal RBCs by macrophages treated with SIRPα-blocking antibodies, the CD47/SIRPα system of cell clearance was suggested to play role in anemia of Rh_null_ individuals, given that expression of CD47 on normal RBCs is coupled to Rh antigen complex (Oldenborg et al., 2000). Indeed, the involvement of CD47 in anemia was also observed in mild autoimmune hemolytic anemia typical of experimental nonobese diabetes, which was worsened and became lethal in CD47-deficient mice (Oldenborg et al., 2002) and in patients with myelodysplastic syndrome, the degree of anemia was correlated to the levels of CD47 expressed on erythroblasts (Jiang et al., 2013). Furthermore, down-regulation of surface CD47 occurring specifically on platelets was connected to thrombocytopenia of *Escherichia coli* infected-mice (Guo et al., 2009), supporting data suggesting that CD47 participates in cytopenia of different etiological sources through cell depletion processes.

Comprehensibly CD47/SIRPα self-recognition system has been implicated in clearance of apoptotic cells. It was demonstrated that apoptosis of neutrophils and Jurkat cells is followed by decreased expression of surface CD47 (Lawrence et al., 2009; Azuma et al., 2011), while in apoptosis of several other nucleated cell types CD47, initially clustered in lipid rafts, is diffused on the cell surface, reducing its avidity for SIRPα (Lv et al., 2015). In both cases, it is believed that the inhibitory signaling triggered by CD47 via phosphorylation of the SIRPA cytoplasmic tail is discontinued and consequently, apoptotic cells are phagocytized. Therefore, elimination of RBCs contributing to the pathogenesis of malarial anemia could also be a reflex of CD47 changes during the apoptosis process.

Reduction of surface CD47 expression has been observed on RBCs maintained under conditions that induce erythrocytic apoptosis, such as blood storage and hypoxia, as detected by externalization of PS (Stewart et al., 2005; Mao et al., 2011), and there is a consensus that *in vivo* elimination of senescent RBCs, which are marked by deflagration of apoptotic processes (Bosman et al., 2005), is regulated by CD47 (Khandelwal et al., 2007; Burger et al., 2012). Nevertheless, with the exception of studies with CD47-deficient mice and Rh_null_ individuals, those addressing a relationship between RBC CD47 and anemia are limited. Two studies on warm autoimmune hemolytic anemia showed no difference in CD47 expression when comparing RBCs from patients with those from healthy individuals, suggesting that CD47 does not play a role in the development of this pathology, in which autoantibodies are well known to be involved (Ahrens et al., 2006; Barros et al., 2009). However, it has been demonstrated that CD47, as a regulator of RBC clearance, is able to overwhelm phagocytosis mediated by antibody and complement (Oldenborg et al., 2001; Olsson and Oldenborg, 2008). Therefore, the contribution of CD47 to autoimmune anemia, possibly by means of its reorganization on cell surface (not assayed in the above mentioned studies), rather than by its downexpression, cannot be excluded.

In malaria, the role of CD47 has been recently addressed (Banerjee et al., 2015; Ayi et al., 2016). Parasites of the nonlethal strain of *P. yoelii* 17XNL were mostly detected infecting young RBCs expressing high levels of CD47, suggesting that malaria parasites could take advantage of negative signaling of CD47 to avoid early elimination by phagocytosis (Banerjee et al., 2015). The authors of this study also demonstrated that absence of this molecule in deficient mice attenuated the course of parasitemia and notably, converted the lethal infection of *P. yoelii* YM into a nonlethal, self-resolving infection characterized by a very low parasite load (peak of 0.06%). Similar results in a more recent study (Ayi et al., 2016) showed that CD47-deficiency attenuated parasitemia and abolished the development of cerebral malaria in *P. berghei* ANKA-infected mice, as well as *P. falciparum*-pRBC had decreased levels of CD47, a phenomenon that was associated with both *in vitro* and *in vivo* clearance of *P. falciparum*—and *P. berghei*-pRBCs via SIRPα, respectively.

These data raise the possibility that alteration of CD47 expression on apoptotic pRBCs operates as a regulator of malaria parasite clearance. Although elimination of pRBCs has a role in pathogenesis of malarial anemia, contrary to RBC lysis that allows the propagation of merozoites at the end of schizogony, RBC apoptosis could play a beneficial role leading intraerythrocytic-developing parasites to degradation by phagocytosis and consequently reducing parasite burden that directly promotes anemia through RBC lysis. This protective role of erythrocytic apoptosis was previously demonstrated by treating *P. berghei*-infected mice with the drug aurothiomalate, which triggers apoptosis selectively in pRBCs with no effect on intraerythrocytic parasite development (Alesutan et al., 2010). This *in vivo* stimulation of pRBC apoptosis delayed the course of parasitemia and mortality of mice, suggesting that the use of drugs to selectively trigger apoptosis pathways in pRBCs could be a strategy to treat malaria in a manner not propitious to generation of parasite resistance, since drugs would target the host cell and not the parasite.

While CD47 changes on pRBCs could play a protector effect, the same is not expected for nRBCs. As mentioned above, CD47 deficiency is associated with development of anemia as a result of increased susceptibility of CD47^−/−^ RBC to phagocytosis under pathological circumstances. Thus, if on one hand RBC clearance promoted by the absence of CD47 attenuates malaria parasitemia (pRBC), on the other hand it leads to anemia (nRBC), as observed in CD47-deficient mice with *P. berghei* malaria (Ayi et al., 2016). This connection between CD47, nRBC and anemia was also confirmed in a study with wild type mice infected by *P. yoelii* 17XNL that found a decline in the number of nRBCs expressing high levels CD47 when RBC counts were falling. An increase in newly formed RBCs (reticulocytes) that express high levels of CD47 was detected later at the recovery stage (Fernandez-Arias et al., 2016), implicating CD47 in the pathogenesis of malarial anemia through elimination of nRBCs.

Paradoxically, deflagration of apoptosis in nRBCs could play a protective role in malaria infection. It is well-known that apoptotic processes represent an innate mechanism against intracellular parasitism that altruistically drives parasites to degradation by phagocytosis along with host parasitized cells, thereby preventing infection of adjacent cells and pathogen propagation (Williams, 1994). Alternatively, apoptosis could act in the control of parasitemia limiting the availability of non-parasitized targeted cells susceptible to infection, as shown in *P. falciparum in vitro* culture where apoptotic nRBCs were refractory to parasite invasion (Totino et al., 2011). However, this phenomenon offering as a host protective mechanism for parasite burden control in malaria while adversely induces anemia, needs to be better examined.

## Apoptosis and defective erythropoiesis

Besides participating in the pathogenesis of malarial anemia by mediating precocious elimination of nRBC, apoptosis could interfere with the production of new RBCs, as previously reported in severe anemia associated to *Leishmania donovani* infection in golden hamsters (Lafuse et al., 2013) as well as in anemia of chronic disease in patients with rheumatoid arthritis (Papadaki et al., 2002), in which cytokine-mediated apoptosis of erythroid cells seems to operate.

Anemia in both malaria experimental models and human infection is followed by an unexpected frequency of peripheral reticulocytes considering the loss of RBCs, indicating that impaired erythropoietic activity is an important mechanism in the pathogenesis of malarial anemia (Chang et al., 2004; Leowattana et al., 2008; Fendel et al., 2010; Anyona et al., 2012; Thawani et al., 2014). Infection by *P. chabaudi, P. yoelii*, and *P. berghei* was shown to inhibit development of erythroid precursors induced by erythropoietin in mice (Thawani et al., 2014), an effect also achieved by *in vitro* incubation of human erythroid cells with hemozoin or proinflamatory cytokines such as TNF and IFN-γ, whose levels are augmented during malaria (Means and Krantz, 1991; Casals-Pascual et al., 2006; Awandare et al., 2011). In addition, in a study with children presenting several degrees of malarial anemia, ineffective erythropoiesis was associated with the amount of circulating hemozoin and TNF, and deposition of hemozoin in bone marrow was related to abnormal erythroid development in postmortem analysis of children with severe anemia (Casals-Pascual et al., 2006).

In this context, proapoptotic effects of hemozoin, TNF and interferon-γ on erythroid precursors have been described *in vitro* (Dai and Krantz, 1999; Lamikanra et al., 2009; Vittori et al., 2010) and high levels of apoptotic erythroid cells were observed in bone marrow aspirates of severe malaria patients presenting erythroid hypoplasia and low percentage of peripheral reticulocytes (Anantrakulsil et al., 2005). Nevertheless, the capacity of both TNF and hemozoin to depress erythropoiesis by inducing apoptosis has not been consensually demonstrated *in vitro* (Skorokhod et al., 2010; Awandare et al., 2011) and apoptosis was not implicated in impaired maturation of erythroblasts during anemia of experimental *P. chabaudi* infection (Chang et al., 2004).

These apparently contradictory results could be related to the differences between cell lines, since *in vitro* studies with hemozoin and TFN were performed with cells from different sources, i.e., erythroid cell lines or fresh isolated CD34^+^ stem cells from PBMC, which have been shown to exhibit different susceptibility to apoptosis induced by TNF or hemozoin (Lamikanra et al., 2009; Skorokhod et al., 2010; Vittori et al., 2010; Awandare et al., 2011). For instance, erythroid cells derived from human erythroleukaemia K562 cell line were susceptible to apoptosis induced by TNF, but not hemozoin, while those cells derived from normal peripheral blood CD34^+^ cells were only susceptible to hemozoin-induced apoptosis, suggesting that these compounds interfere in the development of erythroid cells through distinct pathways, with hemozoin inducing apoptosis and TNF triggering non-apoptotic mechanisms in healthy (non-leukemia) cells. Indeed, a gene expression profile study of purified primary erythroid cultures incubated with either TNF or hemozoin showed recently that both stimuli were able to inhibit erythroid development, with activation of largely distinct transcriptional programs: hemozoin up-regulated genes related to regulation of transcription, cellular stress response and apoptosis, and the majority of genes up-regulated by TNF was involved in innate and adaptive immune responses to infection (Lamikanra et al., 2015).

These data explain, at least in part, the lack of a relationship between apoptosis and depressed erythropoiesis in severe anemia of *P. chabaudi* infection in A/J mice (Chang et al., 2004), with TNF probably overwhelming the proapoptotic property of hemozoin. Although there are no studies concerning the effect of TNF on hemozoin-induced apoptosis, it has been shown that *P. chabaudi* infection in A/J mice is marked by increased levels of TNF at the time of decreased reticulocyte response (Jacobs et al., 1996). Additionally, these data illustrate the well-known concept of the complex multifactorial pathogenesis of malarial anemia, in which the molecular mechanisms regulating eythropoiesis in malaria are still poorly understood; a scenario that reflects a research gap that remains challenging due to practical and ethical difficulties in obtaining bone marrow samples from patients with severe malarial anemia, whose population majority comprises children of African endemic regions.

## Concluding remarks

**Figure 1:**
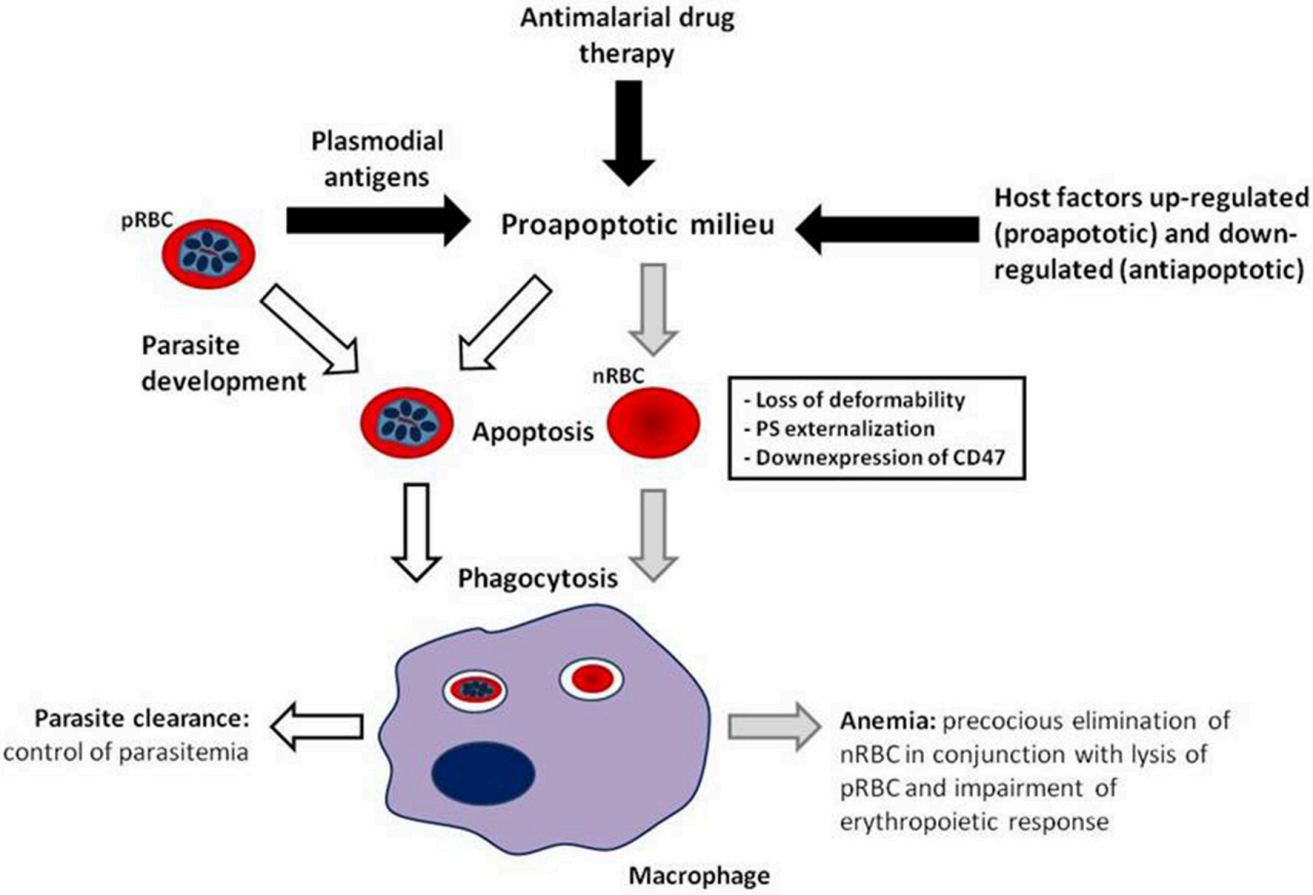
**Model of erythrocytic apoptosis involvement in the pathogenesis of malarial anemia**. Both parasitic antigens and host factors modulated by malaria infection generate a favorable environment for precocious induction of erythrocytic apoptosis (black arrows), which can be enhanced by antimalarial drug treatment, and in the case of parasitized red blood cells (pRBCs), by intraerythrocytic parasite development. Erythrocytic apoptosis provokes cellular changes, i.e., cell rigidity increase, exposure of phosphatidylserine and downexpression of CD47, that drive pRBC (white arrows) and non-parasitized RBC (nRBC; gray arrows) to phagocytosis. Elimination of apoptotic nRBCs along with pRBC lysis and erythropoiesis impairment contribute to the development of malarial anemia. Uptake of apoptotic pRBCs can help control parasite load. In this manner, the pathogenic or protective role of erythrocytic apoptosis in malaria could be a result of increased apoptosis induction differentially in nRBC and pRBC, respectively.

It has become evident that deflagration of cell death processes by apoptosis is not restrict to nucleated cells, occurring also in enucleated RBCs, and consequently, contributing to the development of anemia through precocious elimination of RBCs. In malaria infection, erythrocytic apoptosis occurs and seems to play both detrimental and protective roles by inducing anemia and controlling parasitemia, respectively (Figure 1). Although the magnitude and dynamics of erythrocytic apoptosis in malaria are still to be better established, the above reported connections between apoptosis and anemia open new options for development of therapy for severe malarial anemia by exploring the apoptotic machinery, as considered in other pathologies related to increased induction of apoptosis such as Alzheimer's disease, Parkinson's disease, and sepsis (Jana and Paliwal, 2007; Hattori et al., 2010). Advances that allow therapy improvement for severe malaria anemia are, indeed, urgently needed. The use of adjunctive therapy for severe malarial anemia has not been well explored, possibly due to the common requirement for blood transfusion. However, the prevalence of blood-borne infections such as HIV in malaria endemic areas limits the availability and safety of blood transfusion (Osaro and Charles, 2011). Furthermore, blood transfusion shows a trend toward presentation of severe adverse events and does not significantly decrease mortality rates, underscoring the need for new strategies for treating severe malarial anemia (Meremikwu and Smith, 2000; Obonyo et al., 2007). Thus, inhibition of cell signaling pathways leading to erythrocytic apoptosis could be explored as an alternative strategy to mitigate the impact of severe anemia in mortality caused by malaria.

## Author contributions

PT wrote the manuscript; CD-R and MF critically revised it for intellectual content. All authors read and approved the final manuscript.

### Conflict of interest statement

The authors declare that the research was conducted in the absence of any commercial or financial relationships that could be construed as a potential conflict of interest.
